# The LDL Receptor-Related Protein 1: At the Crossroads of Lipoprotein Metabolism and Insulin Signaling

**DOI:** 10.1155/2017/8356537

**Published:** 2017-05-11

**Authors:** Dianaly T. Au, Dudley K. Strickland, Selen C. Muratoglu

**Affiliations:** ^1^Center for Vascular and Inflammatory Diseases, University of Maryland School of Medicine, Baltimore, MD, USA; ^2^Department of Surgery, University of Maryland School of Medicine, Baltimore, MD, USA; ^3^Department of Physiology, University of Maryland School of Medicine, Baltimore, MD, USA

## Abstract

The metabolic syndrome is an escalating worldwide public health concern. Defined by a combination of physiological, metabolic, and biochemical factors, the metabolic syndrome is used as a clinical guideline to identify individuals with a higher risk for type 2 diabetes and cardiovascular disease. Although risk factors for type 2 diabetes and cardiovascular disease have been known for decades, the molecular mechanisms involved in the pathophysiology of these diseases and their interrelationship remain unclear. The LDL receptor-related protein 1 (LRP1) is a large endocytic and signaling receptor that is widely expressed in several tissues. As a member of the LDL receptor family, LRP1 is involved in the clearance of chylomicron remnants from the circulation and has been demonstrated to be atheroprotective. Recently, studies have shown that LRP1 is involved in insulin receptor trafficking and regulation and glucose metabolism. This review summarizes the role of tissue-specific LRP1 in insulin signaling and its potential role as a link between lipoprotein and glucose metabolism in diabetes.

## 1. Introduction

The metabolic syndrome is comprised of interrelated physiological, metabolic, and biochemical risk factors for type 2 diabetes and cardiovascular disease (CVD). It is well established that the metabolic syndrome correlates with a sedentary lifestyle and obesity, and the syndrome is escalating as a worldwide public health concern due to its prevalence and burden. Furthermore, individuals with the metabolic syndrome have a five-fold increased risk for type 2 diabetes and are twice as likely to develop CVD over the next five to ten years [1]. The metabolic syndrome is currently diagnosed in individuals who present three of the five following risk factors: elevated waist circumference, as defined by population- and country-specific criteria, elevated triglycerides (TGs), reduced high-density lipoprotein cholesterol (HDL-C), elevated blood pressure, and elevated fasting glucose [1]. Insulin resistance has been widely discussed as a potential linking factor for the metabolic syndrome; however, the pathogenesis is unclear and further research is needed to establish a linkage among risk factors.

Type 2 diabetes, a clinical outcome of the metabolic syndrome and the most common form of diabetes, is a complex group of heterogeneous metabolic disorders that includes insulin resistance. The etiology of diabetes is multifactorial and can consist of both genetic and environmental factors, such as low physical activity levels, poor diet, and excess body weight [2]. A metabolic disorder that commonly accompanies diabetes is dyslipidemia, which consists of postprandial lipemia, increased plasma very low-density lipoprotein (VLDL), increased small dense low-density lipoprotein (LDL), and reduced high-density lipoprotein (HDL) [3]. It is speculated from the results of several studies (reviewed in [3]) that diabetic dyslipidemia is caused by several factors, including the effects of insulin on liver apoprotein production, peripheral effects of insulin on adipose and muscle, lipoprotein lipase (LPL) regulation, and effects of cholesteryl ester transfer protein (CETP). This abnormal lipid metabolism is likely a large contributor to the increased risk of atherosclerosis and CVD in diabetics. In addition to CVD, diabetes is also associated with microvascular and macrovascular pathologies, including retinopathy, nephropathy, and neuropathy [4].

The low-density lipoprotein receptor-related protein 1 (LRP1) is a member of the low-density lipoprotein receptor (LDLR) family and is highly expressed in hepatocytes, adipocytes, neurons, vascular smooth muscle cells, fibroblasts, and macrophages. LRP1 is essential for embryonic development [5, 6] and plays a role in the recruitment and maintenance of mural cells during angiogenesis [6]. LRP1 was originally identified as an endocytic receptor for *α*_2_-macroglobulin- (*α*_2_M-) proteinase complexes [7, 8] and apolipoprotein E (apoE) [9], and studies now reveal that LRP1 can bind numerous, unrelated ligands with high affinity (summarized in [10, 11]). In addition to its endocytic function, LRP1 also functions in signal transduction pathways and can interact with other cellular receptors. Several studies have demonstrated that LRP1 is atheroprotective and can regulate processes involved in vascular integrity (reviewed in [12]). More recently, research has shown that LRP1 is involved in insulin signaling and glucose homeostasis in several different tissues. This review briefly summarizes the growing body of literature on LRP1, its role in lipoprotein and glucose metabolism, and its potential influence on the metabolic syndrome and diabetes.

## 2. LRP1 Structure and Mode of Ligand Binding

LRP1 is a large receptor consisting of a 515 kDa heavy chain (*α* subunit) and a noncovalently associated 85 kDa light chain (*β* subunit). As a member of the LDLR family, LRP1 contains modular structures that are shared by all family members and includes cysteine-rich complement-type repeats (CR), epidermal growth factor- (EGF-) like repeats, *β*-propeller (YWTD repeat) domains, a transmembrane domain, and an intracellular domain (ICD) (reviewed in [10, 13]). CR, commonly referred to as ligand-binding repeats, occur in four clusters (termed I–IV) on the LRP1 heavy chain. Interactions between LRP1 and many of its known ligands have been mapped to these clusters. The LRP1 EGF repeats and *β*-propeller domains are predicted to be involved in ligand uncoupling and are located between CR clusters. The LRP1 light chain contains a short extracellular segment, a single-pass transmembrane domain, and an ICD, which contains two dileucine (LL) motifs and two NPxY motifs.

In a canonical mode of ligand binding, ligand recognition occurs when a lysine *ε*-amino group on the ligand forms salt bridges with aspartic acid carboxylates on LRP1 CR. The aspartic acid residues are coordinated and stabilized by a calcium ion and form an acidic pocket. Ligand binding is further strengthened by van der Waals interactions between CR aromatic residues and the aliphatic portion of the ligand lysine residue docked within the acidic pocket. Weak electrostatic interactions can also occur between other ligand lysine residues and additional acidic residues on the CR. The ICD of LRP1 contains two NPxY motifs which can be phosphorylated by activated protein-tyrosine kinases. Both the unphosphorylated and phosphorylated states of the NPxY motifs can serve as a binding site for other proteins [14].

Due to its ability to bind to numerous ligands, LRP1 associates with a chaperone termed the receptor-associated protein (RAP) which prevents premature ligand binding during receptor trafficking from the endoplasmic reticulum (ER) to the Golgi. RAP is a 39 kDa ER-associated chaperone that binds tightly to LRP1 and enables delivery of the receptor to the plasma membrane. Because it binds to LRP1 with high affinity, RAP is frequently used as a competitive inhibitor of LRP1 ligand binding and can inhibit LRP1 receptor function [15, 16].

## 3. LRP1 Single-Nucleotide Polymorphisms (SNPs) Are Associated with Several Diseases

Advances in genome-wide association studies (GWAS) have revealed that LRP1 SNPs are associated with several diseases, including coronary heart disease [17], abdominal aortic aneurysm [18, 19], and migraines [20, 21]. In a recent study by Delgado-Lista et al. [22], top SNPs affecting carbohydrate metabolism were identified in subjects with the metabolic syndrome (European Union LIPGENE project cohort). An LRP1 SNP, rs4759277, was found to be highly linked to several phenotypic features of carbohydrate metabolism, including fasting insulin, C-peptide, HOMA-IR, and QUICKI. Many of the LRP1 SNPs are located within introns, and the effect of these polymorphisms on LRP1 expression and function is currently unknown.

## 4. Glucose Transporters

In most mammalian cells, glucose transport occurs by the process of ATP-independent facilitative diffusion and is mediated by members of the GLUT (SLC2A) family of membrane transport proteins. Fourteen GLUT proteins are encoded by the human genome and include transporters that bind nonglucose substrates, such as fructose, myoinositol, and urate [23]. Although GLUT1–4 are well studied, primary substrates are unknown for at least half of the GLUT family. GLUT proteins contain approximately 500 amino acids with twelve transmembrane *α*-helices and a single N-linked oligosaccharide [24]. GLUT family members are divided into three subclasses based on sequence similarity [25]. GLUT1–4, which are abundantly found in the central nervous system (CNS), pancreatic *β*-cells, hepatocytes, neurons, adipocytes, and skeletal muscles, are briefly discussed below.

### 4.1. GLUT1

GLUT1 is the predominant GLUT isoform expressed in human pancreatic islets [26–28], and glucose sensing in pancreatic *β*-cells are dependent on glucokinase activity (reviewed in [29, 30]). GLUT1 is also the most abundant glucose transporter in the CNS with widespread distribution in the brain [31]. As the most metabolically active organ, the brain depends on capillary bed transport of glucose from the blood to the brain. In contrast to peripheral tissue, glucose transport across the blood-brain barrier (BBB) into the CNS is not dependent on insulin. This process is mediated by GLUT1 [32], which is located in endothelial cells at the BBB. In addition to its function at the BBB, GLUT1 also functions in glial-mediated uptake of glucose from brain interstitial fluid.

### 4.2. GLUT2

GLUT2 is highly expressed in the liver, intestine, and kidney and is expressed at lower levels in the CNS, neurons, astrocytes, and tanycytes [33]. Although GLUT2 is highly expressed in rodent pancreatic islets and is the primary glucose sensor and transporter, GLUT2 is expressed at low levels in human islets [26, 27]. GLUT2 has a low affinity for glucose (*K*_m_ ~17 mM), but its high expression level in select tissues ensures rapid glucose equilibration between the extracellular space and cell cytosol. In pancreatic *β*-cells, GLUT2 is involved in glucose-stimulated insulin secretion (GSIS), and impaired GSIS in diabetic rats was shown to be associated with decreased GLUT2 expression [34, 35]. In the liver, GLUT2 functions to uptake glucose in the fed state and release glucose in the fasting state. Hepatic glucose uptake inhibits glycogenolysis and stimulates glycogen synthesis. Interestingly, GLUT2 in the brain regulates several cellular and physiological functions, including feeding initiation and termination, glucagon secretion, thermoregulation, and the melanocortin pathway (reviewed in [33]). GLUT2 is also involved in autonomic nervous activity and taste preference.

### 4.3. GLUT3

In addition to GLUT1, GLUT3 is the other predominant GLUT isoform expressed in human pancreatic islets [26, 27]. GLUT3, often referred to as the neuron-specific glucose transporter, is also expressed in the brain, testis, spermatozoa, and lymphocytes in humans [23, 31]. Levels of GLUT3 expression in different regions of the brain directly correlate with regional cerebral glucose utilization. GLUT3 has a high affinity for glucose (*K*_m_ ~1.5 mM) and high turnover rate, resulting in efficient glucose uptake in neurons.

### 4.4. GLUT4

GLUT4 is highly expressed in adipose tissue and skeletal muscle and is critical to whole-body glucose homeostasis. The expression and translocation of GLUT4 are highly regulated by insulin, and disruption of this regulatory process results in insulin resistance and an increased risk for developing diabetes (reviewed in [36]). Interestingly, transgenic mice expressing high levels of GLUT4 in adipose tissue or skeletal muscle are highly glucose tolerant and insulin sensitive. Currently, the structure of GLUT4, the cellular components involved in its trafficking, and the insulin-mediated signaling pathways regulating its trafficking are unclear. Recent studies have identified several components of GLUT4 containing vesicles (discussed below), but additional studies are needed to elucidate how each of these components affects GLUT4 function.

## 5. Insulin Enhances Postprandial Lipoprotein Clearance via LRP1 Translocation to the Cell Surface in Hepatocytes

LRP1 is highly expressed in hepatocytes where it functions to mediate the endocytosis and degradation of chylomicron remnants as well as VLDL, *α*_2_M-proteinase complexes, serpin-enzyme complexes, and blood coagulation factor VIII (reviewed in [10]). The binding of chylomicron remnants to LRP1 is mediated by apoE and further enhanced by LPL [37], which remains attached to triglyceride-rich lipoproteins (TRLs) following lipolysis and facilitates hepatic clearance from the circulation [38]. It was recently shown that apolipoprotein C-III (apoC-III), which is highly correlative to plasma TG levels, inhibits the hepatic clearance of TRLs by both LRP1 and LDLR [39].

Early studies found that LRP1 is also abundant in adipocytes [40] and its endocytic function increases 2-3-fold following exposure of the cells to insulin as indicated by increased uptake of ^125^I-labeled activated *α*_2_M (*α*_2_M^∗^) [41, 42]. These findings raised the possibility that insulin may also stimulate LRP1-specific uptake of chylomicron remnants in the liver. To test this hypothesis, Laatsch et al. [43] investigated the effect of insulin on hepatic LRP1-mediated uptake of postprandial chylomicron remnants. Their studies revealed that like adipocytes, insulin stimulated LRP1 translocation to the cell surface in human hepatic HuH7 tumor cells, a rat hepatoma cell line (FAO cells), as well as primary hepatocytes isolated from wild-type (WT) and low-density lipoprotein receptor knockout (LDLR^−/−^) mice.

These results were replicated in vivo by demonstrating that glucose-injected mice showed a significant increase in the hepatic uptake of *α*_2_M^∗^ compared to NaCl-injected control mice [43]. Enhanced uptake of *α*_2_M^∗^ upon insulin stimulation was not observed in leptin-deficient obese mice (ob/ob), which exhibit glucose intolerance and insulin resistance. To confirm the role of LRP1 in the insulin-stimulated hepatic uptake of postprandial chylomicrons, hepatic LRP1 expression was ablated by injecting LRP1^flox^ mice with an adenovirus expressing Cre recombinase. Following intraperitoneal glucose injection, chylomicron remnant uptake was significantly reduced in hepatic LRP1 knockdown animals compared to that in control animals injected with an adenovirus expressing EGFP. Together, these studies demonstrate that insulin stimulates hepatic LRP1 translocation to the cell surface resulting in enhanced chylomicron remnant uptake, thus connecting insulin-mediated signaling events to postprandial lipoprotein catabolism.

## 6. Hepatic Inactivation of LRP1 Impairs Insulin Signaling and Suppresses GLUT2 Translocation to the Plasma Membrane

To further investigate the role of hepatic LRP1 in insulin resistance, mice were generated in which LRP1 was specifically deleted in the liver (h-LRP1^−/−^) [44]. When placed on a high-fat diet (HFD), h-LRP1^−/−^ mice exhibited an accelerated body weight gain attributed to an increase in fat mass, glucose intolerance, pyruvate intolerance, insulin resistance, and dyslipidemia. Furthermore, the mice developed nonalcoholic steatohepatitis. To define potential mechanisms by which h-LRP1^−/−^ mice develop insulin resistance, components of the insulin signaling pathway were investigated. These studies revealed that hepatic LRP1 inactivation resulted in defective insulin signaling, which included impaired phosphorylation of insulin receptor (IR), AKT, and GSK3*β* and incomplete suppression of gluconeogenic genes. Interestingly, h-LRP1^−/−^ hepatocytes had significantly lower levels of IR expression at the cell surface; however, the extent of insulin-stimulated IR internalization was similar between h-LRP1^−/−^ and h-LRP1^+/+^ hepatocytes. These results suggest that efficient IR expression at the cell surface is LRP1 dependent.

Similar to the studies conducted by Laatsch et al. [43], insulin treatment of primary hepatocytes isolated from WT mice stimulated LRP1 translocation to the cell surface. Interestingly, LRP1 translocation was inhibited by the saturated fatty acid palmitate. In contrast, oleic and linoleic acids, unsaturated fatty acid components of the HFD, did not inhibit insulin-stimulated LRP1 translocation. Insulin also increased GLUT2 translocation to the plasma membrane in WT hepatocytes, and in the presence of palmitate, GLUT2 levels in the plasma membrane were further increased. Insulin failed to mediate GLUT2 translocation to the plasma membrane in h-LRP1^−/−^ hepatocytes, suggesting a critical role for LRP1 in insulin-mediated GLUT2 translocation.

These studies provide further evidence that hepatic LRP1 is important for maintaining insulin sensitivity and preventing diet-induced steatosis by regulating insulin signaling and modulating GLUT2 translocation to the plasma membrane in response to insulin. It is not presently clear how LRP1 regulates the trafficking of GLUT2 containing vesicles; however, substantial work has revealed insights into how LRP1 regulates the trafficking of another glucose transporter, GLUT4 (see below).

## 7. LRP1 Regulates Glucose Homeostasis in Adipocytes

The postprandial function of LRP1 has been largely focused on studies in the liver where LRP1 clears apoE-enriched chylomicron remnants and VLDL from the circulation. An early study by Lossow et al. [45] showed that glucose-fed rats incorporated twice the amount of injected chylomicron [^14^C]cholesterol into adipose tissue as fasted rats. This result suggests that insulin may influence chylomicron uptake in adipose tissue. As mentioned earlier, studies by Descamps et al. [42] revealed that LRP1 is abundantly expressed on adipocytes, and receptor activity is significantly increased when exposed to physiological concentrations of insulin. Upon insulin stimulation, primary adipocytes isolated from rat epididymal fat pads exhibited increased uptake of ^125^I-*α*_2_M^∗^ and apoE-enriched *β*-VLDL; however, uptake of ^125^I-*α*_2_M^∗^ and apoE-enriched *β*-VLDL could be inhibited by the GST-39 kDa fusion protein (i.e., GST-RAP) or anti-LRP1 IgG. Insulin also rapidly increased the number of GST-RAP binding sites on the cell surface, suggesting that LRP1 is not synthesized de novo in response to insulin but rather is rapidly translocated to the cell surface from intracellular stores. In vivo rat studies showed that chylomicron uptake was significantly enhanced in animals that were fasted and subsequently given glucose/insulin compared to animals that were only fasted. Chylomicron uptake was further increased in fed rats given glucose/insulin compared to fasted rats given glucose/insulin. The authors proposed that the significant increase in chylomicron uptake in fed rats may be due to a synergistic effect of insulin-induced LRP1 expression on the cell surface and increased LPL activity due to a fed state. This seminal work by Descamps et al. demonstrates that insulin stimulates LRP1 translocation to the cell surface and increases the uptake of chylomicron remnants in adipose tissue.

Further insight into the role of LRP1 in adipocytes was derived from mice in which LRP1 was specifically inactivated in adipose tissue (ad-LRP1^−/−^) [46]. Adipocyte LRP1 knockout mice displayed delayed postprandial lipid clearance compared to ad-LRP1^+/+^ littermates, and delayed lipid clearance was not attributed to defective LPL expression in ad-LRP1^−/−^ mice. Adipose-specific LRP1 inactivation also produced mice with significantly lower body weights due to a reduction in fat mass. Further histological analysis revealed a reduction in both the size and the number of lipid droplets in white and brown adipocytes from ad-LRP1^−/−^ mice. Despite the reduced body weight, ad-LRP1^−/−^ mice consumed more food and were more susceptible to body fat loss under fasting conditions. Observations revealed that ad-LRP1^−/−^ mice displayed difficulty in maintaining body temperature and had a significant increase in energy expenditure compared to ad-LRP1^+/+^ littermates. The enhanced energy expenditure was correlated to an increase in muscle thermogenesis and is likely a compensatory mechanism for the reduction in brown adipose tissue and body temperature in ad-LRP1^−/−^ mice. Interestingly, ad-LRP1^−/−^ mice had improved glucose tolerance and were resistant to HFD-induced obesity and glucose intolerance. This protective effect in ad-LRP1^−/−^ mice is likely due to a shift in energy metabolism consisting of defective lipid uptake and storage and muscle thermogenesis. Together, these studies show that LRP1 regulates adipocyte energy homeostasis and can influence glucose metabolism and insulin sensitivity.

These early cell-based and animal studies revealed a link between adipose LRP1 and glucose metabolism. Nasarre et al. [47] analyzed LRP1 expression in epicardial and subcutaneous fat from type 2 diabetic and nondiabetic patients. Epicardial fat is a metabolically active fat deposit and has been associated with several components of the metabolic syndrome (reviewed in [48]). LRP1 mRNA and protein expression in epicardial fat were significantly higher in diabetic patients compared to nondiabetic patients; however, LRP1 mRNA and protein expression in subcutaneous fat were similar in both patient populations. Moreover, LRP1 mRNA expression in epicardial fat positively correlated with plasma TG (*R*^2^ = 0.50; *P* = 0.01) and plasma glucose (*R*^2^ = 0.33; *P* = 0.03) levels. The authors noted, however, that diabetic patients had a higher, but nonsignificant, incidence of atherosclerosis which may be a confounding factor in the upregulation of LRP1. Results from this patient study suggest that overexpression of epicardial LRP1 may play an important role in the alterations of lipid metabolism associated with T2D.

Collectively, these studies illustrate the complex interplay between insulin signaling and adipose LRP1. Additional studies are needed to determine if the LRP1-mediated uptake mechanism is altered in insulin-resistant individuals and whether altered LRP1 function further influences glucose metabolism and insulin sensitivity in patients with T2D.

## 8. LRP1 Is a Major Component of GLUT4 Containing Vesicles

The mechanism by which LRP1 is translocated to the cell surface following insulin exposure was not fully understood until LRP1 was identified as one of the most abundant proteins in GLUT4 containing vesicles by proteomic analysis [49] (see below). GLUT4 cell surface levels are regulated by insulin, and upon insulin exposure, glucose uptake is substantially enhanced due to the rapid translocation of GLUT4 containing vesicles to the plasma membrane. In adipocytes cultured under basal conditions, 99% of the total GLUT4 is located within the cell [50]. In contrast, approximately 40% of the total GLUT4 is present at the cell surface in insulin-stimulated cells [50].

To identify the proteome of GLUT4 containing vesicles, Jedrychowski et al. [49] developed a unique purification protocol involving differential immunoadsorption of vesicles isolated from light microsomal fractions of primary rat adipocytes. The purified GLUT4 containing vesicles were demonstrated to be sensitive to insulin-mediated translocation and were subjected to semiquantitative and quantitative proteomic analysis. Using this approach, Jedrychowski et al. identified LRP1 and several other membrane proteins as components of these vesicles and confirmed that like GLUT4, LRP1 is translocated to the cell surface in rat epididymal adipocytes, which is consistent with earlier studies [41, 42].

## 9. LRP1 Forms a Complex with Sortilin, IRAP, and GLUT4 and Regulates GLUT4 Trafficking

To identify interacting proteins, the light microsomal fraction isolated from rat epididymal adipocytes was subjected to crosslinking studies [49]. Immunoprecipitation of fraction extracts with anti-GLUT4 IgG identified not only GLUT4 but also LRP1, insulin-regulated aminopeptidase (IRAP), and sortilin, suggesting that these proteins associate as a complex. To determine the impact of LRP1 expression on GLUT4 vesicle formation, LRP1 was silenced in 3T3-L1 adipocytes. Expression of IRAP, sortilin, and GLUT4 decreased in LRP1-silenced adipocytes; furthermore, the LRP1-depleted 3T3-L1 adipocytes demonstrated an approximately 50% decrease in insulin-stimulated glucose uptake. The in vivo impact of LRP1 expression on GLUT4 levels was confirmed by immunoblotting epididymal fat tissue from adipose-specific LRP1 knockout mice, which revealed a decrease in both sortilin and GLUT4 levels. Interestingly, coprecipitation experiments using the LRP1 cytoplasmic domain, prepared as a fusion protein with GST, demonstrated an association with Tbc1D4 (also known as Akt substrate of 160 kDa or AS160). Tbc1D4/AS160 contains two phosphotyrosine binding domains (PTB) and regulates GLUT4 trafficking by catalyzing the hydrolysis of Rab-bound guanosine triphosphate. The two PTB domains in Tbc1D4/AS160 are phosphotyrosine-independent, indicating that they recognize the NPxY motif that is not tyrosine phosphorylated [51]. LRP1 contains two NPxY motifs within its cytoplasmic domain, and tyrosine 4507 is phosphorylated by Src family kinases [52]. Interestingly, Bilodeau et al. [53] discovered that LRP1 is tyrosine phosphorylated in mouse livers following insulin injection, and how this impacts the association of LRP1 cytoplasmic domain with Tbc1D4/AS160 remains to be determined.

## 10. Differentiation of 3T3-L1 Cells into Adipocytes Reduces LRP1 Recycling Rate

By employing quantitative flow cytometric assays, Brewer et al. [54] determined the endocytic rate constant, exocytic rate constant, and fraction of molecules located in the plasma membrane for GLUT4, transferrin receptor, and LRP1. These experiments were performed in 3T3-L1 cells, a murine fibroblast-like cell line that can be differentiated into an adipocyte-like cell line. The results for LRP1 are summarized in Table 1 as *t*_1/2_ values along with additional data from the literature. Under basal conditions, the LRP1 endocytic rate in 3T3-L1 cells is greater than the recycling rate. The net effect is that only 22% of total functional LRP1 is located on the cell surface. Furthermore, the results revealed that insulin has little impact on LRP1 endocytosis and recycling in 3T3-L1 cells. In contrast, upon differentiation into adipocyte-like cells, the LRP1 endocytic rate is slowed by 1.5-fold while the recycling rate is slowed by 4.8-fold, with the combined effect resulting in a 68% reduction of LRP1 surface levels. Interestingly, treatment with insulin increased both the endocytic rate and the recycling rate, resulting in increased levels of LRP1 on the cell surface. Interestingly, knockdown of Tbc1D4/AS160 accelerated LRP1 exocytosis under basal (2.5-fold) and insulin-stimulated (1.6-fold) conditions [54], confirming the functional importance of the interaction of Tbc1D4/AS160 with LRP1.

## 11. A Link between Alzheimer's Disease and Glucose Transport across the Blood-Brain Barrier

Alzheimer's disease (AD) is a neurodegenerative disorder resulting in dementia that affects a large number of the elderly and is characterized by amyloid plaque deposits made up of aggregates of misfolded amyloid *β* (A*β*) oligomers and neurofibrillary tangles in the brain [55, 56]. Since numerous studies have concluded that patients with diabetes have an increased risk of developing Alzheimer's disease (AD) compared to healthy individuals [57–59], the interaction between insulin signaling and the CNS has received significant attention in the last decade. In the CNS, insulin has a well-established role as a growth factor, including effects on synaptogenesis and nerve growth. Furthermore, insulin signaling is crucial for synaptic plasticity, learning, and memory (reviewed in [60]), and thus, neuronal insulin receptor dysfunction could lead to cognitive decline [61, 62].

To examine the impact of glucose transport across the BBB on the progression of AD, elegant experiments were performed by Winkler et al. [32] who employed a mouse model of AD and demonstrated that GLUT1 deficiency intensifies A*β* peptide accumulation in the brain of these mice. While blood glucose levels were unchanged in GLUT1^−/−^ mice, cerebrospinal fluid (CSF) glucose levels were dramatically reduced. Interestingly, a significant reduction in capillary length in the somatosensory cortex and hippocampus were noted in GLUT1^−/−^ mice resulting in reduced blood flow. The study also demonstrated that lower levels of GLUT1 lead to diminished levels of LRP1 expression, further aggravating A*β* accumulation as LRP1 plays a major role in clearing this peptide. Upon rescue of GLUT1 expression, LRP1 protein levels normalized in the brain capillaries of the AD mouse model, demonstrating a connection between GLUT1 and LRP1 expression levels. This effect of GLUT1 on LRP1 expression was connected to increased levels of the sterol regulatory element binding protein 2 (SREBP2), which is a known transcriptional suppressor of LRP1 [63].

## 12. Neuronal LRP1 Regulates Glucose Metabolism

To determine a role of LRP1 on neuronal glucose metabolism, Liu et al. [64] generated a forebrain neuron-specific LRP1 knockout (n-LRP1^−/−^) mouse and observed that the insulin receptor *β* (IR*β*) and phosphorylated Akt levels were significantly decreased in n-LRP1^−/−^ mice compared to those in control mice. These results were consistent with cell-based studies in which LRP1 expression was knocked down. Coimmunoprecipitation experiments confirmed that LRP1 interacts (directly or indirectly) with the IR*β*. Importantly, cellular glucose uptake levels were demonstrated to be impaired in LRP1-deficient cells. The levels of GLUT3 and GLUT4 were reduced in primary neurons in which LRP1 was silenced, as were the levels of these two glucose transporters in the cortex of n-LRP1^−/−^ mice. Finally, the study demonstrated that LRP1 deficiency in neurons leads to glucose intolerance in the brain. Together, these studies revealed that LRP1 modulates insulin signaling in neurons similar to its role in hepatocytes.

Prior studies have shown that insulin treatment alters intracellular trafficking of LRP1, stimulating recycling of LRP1 to the plasma membrane in both adipocytes [41] and hepatocytes [43]. To determine if this occurs in neurons, SH-SY5Y human neuronal cells were treated with insulin, and the results confirmed enhanced surface expression of LRP1 upon insulin treatment [64]. Furthermore, the study demonstrated that hyperglycemia suppresses LRP1 expression in the brain. Since LRP1 plays an important role in the clearance of the A*β* peptide in the brain, lower levels of LRP1 could exacerbate AD pathology.

## 13. Circulating Lipoprotein Delivery to the Brain Regulates Insulin Signaling in *Drosophila*

Contrary to the earlier dogma that the CNS was not considered to be an insulin-sensitive tissue, the CNS is now regarded as an insulin-sensitive organ. The IR is widely expressed in the brain [65], and IR-mediated signaling promotes neuronal development, glucoregulation, feeding behavior, body weight, cognitive processes, executive functioning, learning, and memory formation [66]. Local insulin production in the CNS in mammals has been under intense debate. While a body of work supports the expression of insulin in the CNS (reviewed in [67]), there is some debate regarding the existence of definitive evidence [68]. Interestingly, in *Drosophila*, three of the seven circulating insulin-like peptides are secreted from the brain [69] and act locally on feeding behavior and systemically to regulate metabolism.

Since insulin and its signaling pathway have been well conserved over the course of evolution, Brankatschk et al. [70] used a *Drosophila melanogaster* model for investigating insulin signaling in neurons and discovered that the BBB is the main sensor to report the nutritional status in these organisms. Surprisingly, it is not the total calories absorbed, but rather the lipid composition of consumed food that is sensed by special neurons which in turn regulate insulin signaling.

In *Drosophila*, the fat body is an organ analogous to the liver and produces lipophorin, the major hemolymph (vertebrate blood analog) lipid carrier. In addition, the fat body also produces lipid transfer particle (LTP) which is responsible for transferring lipids onto lipophorin. Brankatschk et al. [70] discovered that LTP can cross the BBB in a process mediated by LRP1 and LRP2 (megalin). Furthermore, they found that fat molecules derived from yeast-based food (as opposed to those derived from plant-based food) promoted LTP accumulation in the brain which enhanced insulin signaling and promoted fly larvae growth. These studies revealed that in *Drosophila*, fat-containing molecules carry specific nutrient information to sensory cells in the brain. This finding opens the intriguing question of whether a generalized mechanism in mammals exists in which lipoproteins carry information from the periphery to the brain to regulate glucose homeostasis.

## 14. Conclusions

**Figure 1:**
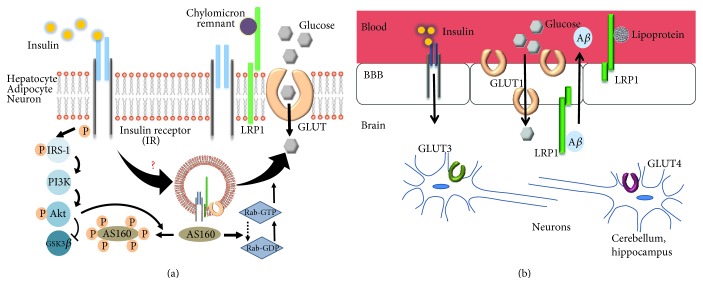
(a) LRP1-dependent insulin signaling in peripheral tissues. Insulin stimulates LRP1 trafficking to the plasma membrane of hepatocytes, adipocytes, and neurons. GLUT2 in hepatocytes, GLUT4 in adipocytes, and GLUT3 and GLUT4 in neurons are translocated to the plasma membrane in an LRP1-dependent manner upon insulin stimulation. Upon binding to its receptor, insulin initiates signaling pathways that mediate GLUT4 translocation to the plasma membrane in a process regulated by LRP1 expressed in GLUT4 containing vesicles. Akt activation by insulin is crucial for this process as it causes AS160 phosphorylation at multiple sites and inactivates its GAP activity. AS160 is known to associate with the LRP1 cytoplasmic domain. Rab GTPase activation in turn stimulates GLUT4 translocation. (b) Glucose and insulin metabolism is modulated by LRP1 in the brain. Insulin production by cells in the brain is somewhat controversial, although insulin is delivered from the blood to the brain by receptor-mediated transcytosis. The insulin receptor rarely induces glucose uptake by brain cells. Instead, it has effects on feeding that are largely opposite to those produced by insulin in the periphery. CNS insulin also impacts cognition. In the brain, the most metabolically active organ, glucose acquisition is independent of insulin, and glucose transporter proteins (GLUTs) mediate glucose delivery from the blood to the brain through the blood-brain barrier (BBB). GLUT1 is detected exclusively in the endothelial cells of the BBB as well as all other neural cells (in a distinct molecular form different than BBB). GLUT3 is specifically expressed in neurons. GLUT4 is an insulin-sensitive transporter and is only expressed at lower levels in specialized neurons of the hippocampus and the cerebellum. The function of the BBB-localized LRP1 in actively removing A*β* from the brain is regulated by insulin levels. Through the endocytic function of LRP1, the BBB may act as the main sensor to report the nutritional status, especially the lipid composition of consumed food to special neurons which in turn regulate insulin signaling, as it was reported to be the case in *Drosophila*.

The initial observations that LRP1 cell surface levels and functional activity increased in response to insulin treatment led to the proposal that insulin signaling may modulate lipoprotein catabolism [42]. This proposal was proven in subsequent studies where intraperitoneal injection of glucose enhanced LRP1 levels on the hepatic surface and increased the uptake of chylomicron remnant lipoprotein particles [43]. Additional work revealed that LRP1 directly regulates the insulin signaling pathway (Figure 1(a)) [44, 64], although the molecular mechanism of how this occurs is not yet clear and requires further investigation. Studies have also identified LRP1 as a major component of GLUT4 containing vesicles and have further shown that cellular trafficking of these vesicles depends on LRP1 expression [49]. Again, the molecular mechanism is largely unknown and will require additional studies. Whether LRP1 is contained in GLUT2 containing vesicles in the liver and can modulate vesicle trafficking remains to be established. Finally, studies in the brain employing mouse models suggest a connection between GLUT1 expression levels and LRP1 expression levels [32], but it is presently unknown how these two molecules are linked. Interestingly, studies performed in *Drosophila* [70] revealed that fat-containing molecules absorbed from the diet carry specific nutrient information to sensory cells in the brain. This finding raises the intriguing question of whether a generalized mechanism in mammals exists in which lipoproteins carry information from the periphery to the brain to regulate glucose homeostasis. As the current debate continues on whether AD represents a form of diabetes (type 3 diabetes) that selectively afflicts the brain, LRP1's protective role against neurodegenerative disease is now highlighted as a molecule converging the roles of removing excess A*β* peptide from the brain, lipoprotein metabolism, and mediating insulin signaling (Figure 1(b)).

## Figures and Tables

**Table 1:** *t*_1/2_ values calculated from rate constants for LRP1 recycling in cells.

Ligand	Cell	Condition	*t*_1/2_ endocytosis (min)	Fold	*t*_1/2_ exocytosis (min)	Fold	^a^PM	Fold	Ref
AF647-*α*_2_M	3T3-L1 cells	Basal	1.69	1.0	6.93	1.0	0.22	1.0	[54]
AF647-*α*_2_M	3T3-L1 cells	Insulin	1.69	1.0	6.30	0.9	0.24	1.1	[54]
AF647-*α*_2_M	3T3-L1 differentiated	Basal	2.48	1.5	33.00	4.8	0.07	0.3	[54]
AF647-*α*_2_M	3T3-L1 differentiated	Insulin	2.04	1.2	16.12	2.3	0.11	0.5	[54]
^125^I-8G1	WI-38 fibroblasts	Basal	4.81	2.8	ND	ND	ND	ND	[71]
^125^I-*α*_2_M	Human SMCs	Basal	1.08	0.6	ND	ND	ND	ND	^b^
^125^I-*α*_2_M	CHO LRP1 null transfected with chicken LRP1	Basal	2.68	1.6	ND	ND	ND	ND	[72]

^a^Fraction of total expressed in plasma membrane [54]. ^b^M. Migliorini, S. C. Muratoglu, D. T. Au, and D. K. Strickland, unpublished data. ND: not determined.
